# Acceptability and Feasibility of Integrating Point-of-Care Diagnostic Testing of Sexually Transmitted Infections into a South African Antenatal Care Program for HIV-Infected Pregnant Women

**DOI:** 10.1155/2018/3946862

**Published:** 2018-05-09

**Authors:** E. Morikawa, M. Mudau, D. Olivier, L. de Vos, D. Joseph Davey, C. Price, J. A. McIntyre, R. P. Peters, J. D. Klausner, A. Medina-Marino

**Affiliations:** ^1^Division of Infectious Disease, David Geffen School of Medicine, University of California, Los Angeles, Los Angeles, CA, USA; ^2^Research Unit, Foundation for Professional Development, Pretoria, South Africa; ^3^Department of Epidemiology, Fielding School of Public Health, University of California, Los Angeles, Los Angeles, CA, USA; ^4^Department of Epidemiology and Biostatistics, School of Public Health and Family Medicine, University of Cape Town, Cape Town, South Africa; ^5^Anova Health Institute, Johannesburg, South Africa; ^6^School of Public Health and Family Medicine, University of Cape Town, Cape Town, South Africa; ^7^Department of Medical Microbiology, School of Public Health and Primary Care, Maastricht University, Maastricht, Netherlands; ^8^Department of Medical Microbiology, University of Pretoria, Pretoria, South Africa

## Abstract

**Background:**

* Chlamydia trachomatis (CT)*,* Neisseria gonorrhoeae (NG)*, and* Trichomonas vaginalis* (TV) infections may increase the risk of vertical transmission of the human immunodeficiency virus (HIV). In resource-limited settings, symptomatic screening, and syndromic management of sexually transmitted infections (STIs) during pregnancy continue to be the standard of care. In the absence of diagnostic testing, asymptomatic infections in pregnant women go untreated.

**Objective:**

To describe the acceptability and feasibility of integrating diagnostic STI screening into first antenatal care visits for HIV-infected pregnant women.

**Methods:**

HIV-infected pregnant women were recruited during their first antenatal care visit from three antenatal care clinics in Tshwane District, South Africa, between June 2016 and October 2017. Self-collected vaginal swabs were used to screen for CT, NG, and TV with a diagnostic point-of-care (POC) nucleic acid amplification test. Those with STIs were provided treatment per South African national guidelines.

**Results:**

Of 442 eligible women, 430 (97.3%) agreed to participate and were tested. Of those with a positive STI test result (*n* = 173; 40.2%), 159 (91.9%) received same-day results and treatment; 100% of STI-infected women were treated within seven days.

**Conclusions:**

Integration of POC diagnostic STI screening into first-visit antenatal care services was feasible and highly acceptable for HIV-infected pregnant women.

## 1. Background

The high global prevalence of* Chlamydia trachomatis (CT)*,* Neisseria gonorrhoeae (NG)*, and* Trichomonas vaginalis* (TV) continues to impart adverse health outcomes in women. During pregnancy, those infections may result in substantial fetal and neonatal morbidity and mortality [1–6]. Recent research further suggests that, among HIV-infected pregnant women, STIs may increase the risk of mother-to-child transmission (MTCT) of HIV via increased HIV viral load shedding resulting from genital inflammation [7–9].

While some countries recommend universal diagnostic testing for STIs as a component of antenatal care [10], the World Health Organization (WHO) recommends symptomatic screening with syndromic management for STIs in resource-limited settings [1, 10, 11]. However, given that most STIs are asymptomatic, the majority of women remain undiagnosed and untreated for their infection(s) [12]. Physiological changes during pregnancy, such as changes in vaginal discharge and urinary habits, may further mask the signs and symptoms of a true infection.

In accordance with WHO guidelines, South Africa conducts symptomatic screening and syndromic management of STIs during pregnancy [13, 14]. With the goals of reducing STIs during pregnancy and preventing harmful sequelae, including vertical transmission of HIV infection, we sought to assess the acceptability and feasibility of implementing point-of-care (POC) diagnostic STI testing and same-day treatment in antenatal clinics in Tshwane District, South Africa.

## 2. Materials and Methods

### 2.1. Study Design

We evaluated the acceptability and feasibility of integrating POC STI diagnostic screening and same-day treatment into the antenatal care (ANC) services provided by public clinics to HIV-infected pregnant women during their first ANC visit. For this analysis we used data collected from women enrolled into a prospective cohort study investigating the impact of POC diagnostic screening and treatment of CT, NG, and TV on adverse birth outcomes and HIV MTCT [15].

### 2.2. Setting

This study was conducted in three primary healthcare clinics in Tshwane District, South Africa; Tshwane District is home to Pretoria, South Africa's national capital. On average, each clinic provides care to ~138 first ANC patients per month. Standard clinical care offered to all pregnant women attending their first ANC visit in public-sector clinics includes (1) a health education session (i.e., information on the impact of HIV infection, STI, and nutrition on pregnancy), (2) confirmation of pregnancy status on urine specimens, (3) gestational age estimation, (4) HIV testing with a rapid test, and (5) STI screening using a syndromic algorithm as per South African maternity care guidelines [13]. HIV-infected women not currently taking antiretroviral therapy (ART) are initiated on ART during this visit [16]. Women who screen positive for STI-related symptoms are provided treatment per South African STI management guidelines [14].

### 2.3. Definition of Parameters

We assessed the acceptability and feasibility of integrating POC diagnostic screening of CT, NG, and TV and same-day treatment into routine antenatal care services for HIV-infected women. Acceptability was defined as >80% of eligible women consenting to be tested. Feasibility was defined as >90% of women with a positive STI test result receiving same-day treatment per South African national guidelines. A GeneXpert® IV (Cepheid, Sunnyvale, CA, USA) diagnostic testing system was installed in each clinic.

### 2.4. Staff Training

Each site was staffed with one study nurse and one study assistant (herein after collectively referred to as “study staff”). Prior to study implementation, study staff were trained on study protocols, patient consenting and confidentiality, and online data collection systems. Study staff also underwent a 7-hour training session on operating, maintaining, and troubleshooting the GeneXpert system.

### 2.5. Description of Screening Process

POC diagnostic screening for CT, NG, and TV was incorporated into the package of basic care offered to HIV-infected pregnant women during their first ANC visit. Pregnant women diagnosed with HIV infection on the day of, or prior to, their first ANC visit were assessed for eligibility by study staff. Eligibility criteria included age ≥ 18 years, gestational age ≤ 34 weeks, documented HIV infection, and residence in and intention to remain in Tshwane through delivery. Written informed consent was sought from all participants prior to enrollment. Consent forms were translated into local languages and administered in the participant's preferred language.

Sociodemographic and clinical data and contact details were collected from consenting participants by research assistants through an interviewer-administered structured questionnaire. The questionnaire also investigated STI test preferences, including the type of specimen they preferred to have tested (vaginal swab or urine) and specimen collection methods (self-collected or clinician-collected).

All women were tested on self-collected vulvovaginal swab specimens using Xpert® CT/NG Vaginal/Endocervical Specimen Collection Kits (Cepheid, Sunnyvale, CA, USA). CT and NG were tested using the Cepheid Xpert CT/NG assay and TV was tested using the Xpert TV assay. During the 90-minute test, study nurses provided standard, first ANC visit care services per South African national guidelines. Additional genital specimens for repeated testing and/or biobanking were collected from all participants.

### 2.6. Reporting of Test Results and Treatment

Participants were informed of their test results before leaving the clinics. Those who tested positive were immediately provided treatment. Participants who could not wait for their test results were contacted telephonically within 24 hours. Those who tested negative for all three STIs were informed of their result by telephone. Those who tested positive for any of the three STIs were requested to return to the clinic, where they were informed of their results and were provided treatment. All participants were given standard counselling on safer sex practices and STI prevention. Participants who tested positive for an STI were provided a clinic referral slip for their partner(s) and the option to take home treatment pill packet(s) for their partner(s). All participants with a positive test result were requested to return to the clinic between 21 and 28 days after treatment for a follow up test-of-cure.

### 2.7. Ethical Considerations

The institutional review boards of the University of California, Los Angeles and the University of Pretoria provided study protocol approval. Permission was obtained from the district Department of Health and clinic managers.

## 3. Results

### 3.1. Acceptability of Intervention

**Figure 1:**
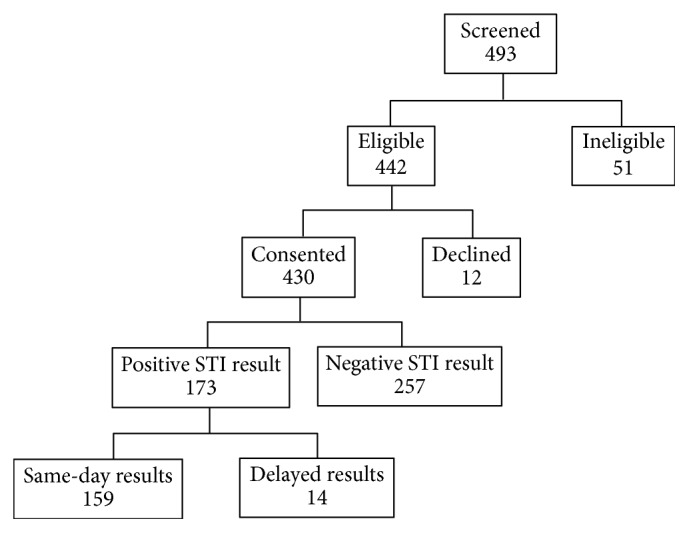
Schematic diagram of the number of women approached, enrolled, and tested in the antenatal screening study in Tshwane District, South Africa (June 2016–October 2017).

A total of 493 women were screened for study participation, of whom 442 (89.7%) met eligibility criteria (Figure 1). Among those 442 eligible women, 430 (97.3%) consented to participate and be tested. When surveyed on the type of specimen they preferred to provide for STI testing, 51 (11.9%) preferred to provide a urinary specimen, 188 (43.8%) preferred to provide a vaginal specimen, and 190 (44.3%) had no preference. The majority of women (64.3%) preferred to self-collect vaginal swab specimens, while 10.5% preferred nurse-collected specimens and 25.2% expressed no preference (Table 1).

### 3.2. Feasibility of Intervention

Of the 430 women tested, 173 (40.2%) had at least one positive STI result (CT = 29.5%; NG = 5.6%; TV = 20.2%) (Table 2). Detailed results on STI prevalence and associated factors have been reported elsewhere [15, 17]. Of the 174 participants with an STI, 173 were treated for their STI by study nurses; one participant with a positive test result had received syndromic management of their STI elsewhere six days prior to study enrollment. Overall 159 (91.9%) of women infected with CT, NG, and/or TV received their results and relevant treatment on the same day of their first ANC clinic visit (CT = 92.9%; NG = 95.8%; TV = 90.8%) (Table 3). All women received results and treatment within 7 days of the visit. Of those with STIs, 163 (94.8%) indicated that they would inform their partner(s) of their test result, of whom 20.5% (33/161) accepted both a partner pill packet and partner referral slip.

Of the 430 women tested, 397 (92.3%) received valid test results after a single Xpert CT/NG test run, 28 (5.5%) received valid test results after a second test run, and 5 (1.3%) required more two than 2 test runs before receiving valid test results (Table 4). For TV, 417/430 (97%) received valid test results after a single Xpert TV test run, 12 (2.8%) received valid test results after a second Xpert TV test run, and only 1 (0.2%) required more than 2 test runs before receiving valid Xpert TV test results (Table 4).

## 4. Discussion

We implemented diagnostic screening and same-day treatment services for three highly prevalent STIs into antenatal clinic services at three busy antenatal care clinic in Tshwane District, South Africa. The high percentage (>97%) of eligible participants consenting to participate and 100% engagement in self-collection of vaginal swab specimens suggest that our intervention was highly acceptable. The ability of study staff to successfully screen and treat >90% of participants within a single clinic visit demonstrated the high feasibility of the intervention.

An important consideration in executing the intervention was the integration of POC STI testing into a woman's first ANC appointment. The intervention was purposefully designed to allow routine ANC clinical services to be rendered while the tests were running and be completed shortly after a routine consultation was completed. Consequently, integrated STI testing did not disrupt clinic flow, nor did it significantly lengthen the general visit time, thus contributing to the feasibility of the study. Furthermore, participants were not financially compensated for enrollment, suggesting that participation was due to the perceived benefits of the intervention, not incentives.

Results from this study align with previous acceptability and feasibility studies of STI screening in antenatal care conducted among pregnant women in low- and middle-income countries. Specifically, Wynn et al. used the Xpert platform and CT/NG and TV assays with self-collected vaginal swabs to successfully implement STI screening and treatment in Gaborone, Botswana, in 2015 [18]. In that study, 89% of eligible women consented to screening, and 100% of the women who tested positive for an STI were treated. Cabeza and colleagues conducted an STI screening study to test for CT among pregnant women in Lima, Peru, in 2013; this group used off-site STI testing, in contrast to point-of-care testing [19]. In Cabeza's study, 94% of eligible pregnant women consented to STI screening and 98% of those who tested positive for STI were treated. Those studies support our hypothesis that integrating effective STI screening into antenatal care services can be successfully implemented in a range of resource-limited settings, even when specimens are tested off-site as in the Peru study.

Recently published data from our study suggests that integration of POC diagnostic screening for STIs into ANC services increases the identification of women with curable STIs who would have been missed if programmes relied solely on the syndromic screening algorithms. Despite that benefit, introduction of the POC technology we used required additional financial, human resource, and infrastructure investments. The testing platform required additional desk space, a reliable source of electricity, and efficient temperature control. We used dedicated trained research nurses, implemented quality control measures, and provided additional assistance, as needed. Studies investigating the cost and cost-effectiveness of integrating POC diagnostic screening for curable STIs into ANC in low-resource settings are urgently needed.

Our study had several limitations. First, our intervention was conducted by study staff carefully instructed and trained on procedures that are currently outside the scope of facility nurses. Thus, implementing routine STI screening and treatment into general ANC clinical practice will require ANC nurses to have the capacity to perform new, additional tasks, including explaining to clients how to collect a vaginal swab specimen and, depending on the site, operating the Xpert testing system. The second limitation was that the Xpert system, while easy to use, requires thorough training on the handling and loading of cartridges, maintenance of the machine, and minor troubleshooting. Lack of sufficient technological and/or human resources in certain settings may make full, seamless integration of the system into a busy clinic difficult to achieve. A final limitation was the lack of standardized assessment surrounding the retrospective perception of the participants, healthcare providers, and clinic managers regarding the integration of STI screening.

In conclusion, we found that integrating diagnostic STI screening of HIV-infected pregnant women during their first antenatal care visit was highly acceptable and feasible in the context of research-supported high-volume ANC clinics in resource-constrained settings. The Xpert test was able to detect STIs in pregnant women in a timely fashion, allowing for same-day treatment and possible prevention of complications in pregnancy.

## Figures and Tables

**Table 1:** Characteristics of the human immunodeficiency virus-infected pregnant women enrolled in the antenatal screening study in Tshwane District, South Africa (June 2016–October 2017).

	*n*	%
Total	430	100%
Age (years), median (IQR)	30	(26–34)
<25 years	71	16.6
25–35 years	287	66.7
>35 years	72	16.7
Relationship with father of baby		
No relationship	15	3.5
Steady partner	187	43.7
Living together	167	38.9
Married	60	13.9
Education level		
Below matric	236	55.0
Matric certificate and above	193	45.0
Gestational age by LNMP at enrollment		
1st trimester (1–12 weeks)	95	22.8
2nd trimester (13–27 weeks)	276	66.4
3rd trimester (≥28 weeks)	45	10.8
Gravidity		
First pregnancy	56	13.0
≥second pregnancy	374	87.0
Preferred STI specimen		
Urine	51	11.9
Vaginal swab	188	43.8
Either	190	44.3
Preferred method of vaginal swab collection		
Self-collected	275	64.3%
Nurse-collected	45	10.5%
Either (no preference)	108	25.2%

Percentages may exceed 100% due to rounding; IQR: interquartile range; LNMP: last normal menstrual period.

**Table 2:** Prevalence of *Chlamydia trachomatis*, *Neisseria gonorrhoeae*, and *Trichomonas vaginalis* among HIV-infected pregnant women in the facilities in Tshwane District, South Africa.

	Positive (*N* = 430)	%
Any STI (CT/NG/TV), *n* (%)	174	40.5%
Any CT infection, *n* (%)	127	29.5%
Any NG infection, *n* (%)	24	5.6%
Any TV infection, *n* (%)	87	20.2%

**Table 3:** Time to treatment among HIV-infected pregnant women who tested positive for CT, NG, and TV in three clinics in Tshwane District, South Africa.

Time to treatment	Any STI (CT/NG/TV)		CT		NG		TV	
	*n*	%	*n*	%	*n*	%	*n*	%
Same day	159	91.9%	117	92.9%	23	95.8%	79	90.8%
1-2 days	11	6.4%	7	5.6%	1	4.2%	7	8.1%
3–5 days	1	0.6%	0	0.0%	0	0.0%	1	1.2%
6-7 days	2	1.2%	2	1.6%	0	0.0%	0	0.0%
>7 days	0	0.0%	0	0.0%	0	0.0%	0	0.0%

**Table 4:** Number of repeat Xpert CT/NG and Xpert TV testing performed on HIV infected pregnant women in three clinics in Tshwane District, South Africa.

	CT/NG		TV	
	*n*	%	*n*	%
No repeated tests	397	92.3%	417	97.0%
1 repeated test	28	5.5%	12	2.8%
2 repeated tests	2	0.6%	1	0.2%
3 repeated tests	3	0.7%	0	0.0%
